# Characterization of Biopolymer Hydrogels Prepared with Water Exposed to Indirect Plasma Treatment

**DOI:** 10.3390/ijms252413427

**Published:** 2024-12-14

**Authors:** Żaneta Król-Kilińska, Dominika Kulig, Anna Zimoch-Korzycka, Edward Reszke, Łukasz Bobak, Slaven Jurić, Andrzej Jarmoluk

**Affiliations:** 1 Department of Functional Food Products Development, The Faculty of Biotechnology and Food Science, Wroclaw University of Environmental and Life Sciences, Chelmonskiego 37/41, 51-630 Wroclaw, Poland; dominika.kulig@upwr.edu.pl (D.K.); anna.zimoch-korzycka@upwr.edu.pl (A.Z.-K.); lukasz.bobak@upwr.edu.pl (Ł.B.); andrzej.jarmoluk@upwr.edu.pl (A.J.); 2 Plasma Investment Ltd., Research and Development Department, Wroclaw Technology Park, 13 Dunska Str., 54-427 Wroclaw, Poland; e.reszke@plasmainvestment.com; 3 Faculty of Agriculture, Division of Agroecology, Department of Chemistry, University of Zagreb, Svetošimunska cesta 25, 10000 Zagreb, Croatia; sjuric@agr.hr

**Keywords:** water, plasma, indirect treatment, micro-clustered, hydrogel, sodium alginate, carrageenan, gelatin, texture

## Abstract

This study aimed to evaluate the influence of indirect-plasma-treated water (IPTW) in the preparation of hydrogels. Three commonly used natural, biodegradable polymers with the ability to form gels were selected: gelatin, carrageenan, and sodium alginate. The pH, gelling temperature, texture profile, swelling degree, and color of hydrogels were evaluated, and the polymers were subjected to Fourier-transform infrared (FTIR) spectroscopy. The morphology of the hydrogels was investigated using Scanning Electron Microscopy (SEM). Additionally, the physiochemical properties of the water media, which were distilled water (DW) and IPTW, were analyzed. The results indicated that the gels prepared using IPTW were characterized by a lower pH, higher hardness and lower gelation temperature. After 48 h of swelling ratio (SR) testing, gelatin and alginate hydrogels made with IPTW were characterized by lower SR, while an inverse relationship was found in the case of SR of carrageenan gels. The FTIR analysis confirmed changes in the water binding ability. The use of IPTW also significantly affected the microstructure of the tested materials. A statistically significant change in the color of IPTW gel samples was also noted. The results showed that IPTW induces physicochemical changes in hydrogels, which can lead to the enhancement of their practical applications.

## 1. Introduction

Hydrogels are three-dimensional network materials with a porous microstructure with high water content resulting from their ability to swell. The properties of hydrogels can vary significantly depending on the type of polymer matrix, the method and degree of crosslinking, and the three-dimensional structure. However, the common feature of all hydrogels is the presence of water as their core component. Many of the physical properties of hydrogels depend on this water content and its organization [1]. The presence of water in hydrogels significantly affects their mechanical properties, which determine their wide range of applications, such as food packaging systems [2,3,4], sensors [5,6], drug delivery technologies [7,8,9], tissue engineering [10,11,12], wastewater treatment [13,14,15], soil stabilization [16,17,18] and supercapacitors [19,20,21]. The greatest advantages of using hydrogels are their degradability, environmental friendliness, low cytotoxicity, etc. [22]. The properties of hydrogels such as absorption, release, transportation, and response to external stimuli are strictly dependent on the hydrogen bond network’s dynamics and structure, and therefore the presence of water [23].

Water is the main component of living organisms, the most abundant liquid on Earth, which participates in a wide range of biological, geological, and chemical processes. The properties of water are complex and characterized by numerous anomalies and unexpected phenomena. Depending on temperature, the structure and chemistry of the surface, pressure, and the presence both of polar and nonpolar solutes and atomic and molecular ions, the water properties may change [24]. Liquid water is a dynamic mixture that forms a three-dimensional hydrogen bond network continually undergoing topological reformation. The properties of water strictly depend on the hydrogen bond network, which may be influenced by many natural forces. The aggregation of water molecules through weak interaction leads to the formation of molecular clusters [25].

Plasma treatment causes the disintegration of larger agglomerates (clusters) of water into smaller ones, affecting its properties. Various terms are used in the context of plasma-treated water, such as plasma-treated water (PTW) [26,27], plasma-activated water (PAW) [28,29], and micro-clustered water (MC) [30,31,32]. Generally, the different naming conventions stem from the distinct physical processes involved in treating water with plasma. Plasma-activated water (PAW) has recently been highlighted as an interesting sanitizer [33,34,35], modification of starch [36], wine quality improver [37], and thawing curing agents for meat, fish and their products [38,39]. It is widely used for washing fruit and vegetables to prevent postharvest spoilage [40,41,42]. Plasma reactors for PAW production can be categorized into three main types: direct discharges in liquids, discharges in the gas phase above a liquid, and discharges within bubbles inside liquids, or in contact with liquid spray mists or foams. The operating principle of these reactors is based on the transfer of ions, electrons, and radicals to the surface of the material being treated. Plasma treatment of liquids can occur through contact or non-contact mechanisms. Generally, PAW is produced using treatment in the contact mode. During the process, free radicals are produced through direct interactions between liquid molecules and the electrons and ions from the plasma [43].

In this study, a novel plasma treatment method for indirect plasma-treated water (IPTW) production [30,44] was employed. In this method, the modification of the liquid structure is made possible by the use of an external factor in the form of electromagnetic noise generated in the wideband range of acoustic as well as electromagnetic waves in noise diodes. It is important to determine the frequency of emitter signals that would coincide with the natural frequencies of clusters or larger structures of molecules of a given liquid, whose self-matching resonant frequencies lie in the noise diode bands. The resonant, self-tuning of the electromagnetic radiation with the frequency of liquid vibrations is the foundation of the process [32]. The intensification of vibrations within water clusters may disrupt the hydrogen bonds connecting ions within the clusters, resulting in structural changes in the water. Unlike other plasma-based water treatment methods, in this novel technique, the transformation energy was not transferred via free radicals. No free radicals are generated during the process, and none are present in the water following production. The process, which involves the generation of ‘cohered’ noise through the presence of excited trace water vapor delivered to the plasma using a dynamic vacuum system in the reactor, is highly energy-efficient and considerably shorter than other known systems. This has enabled the industrial-scale application of the water treatment process [44].

Although there is a significant amount of literature regarding the use of plasma treated water, there is no literature on the use of indirect treated plasma water (IPTW) in the production of hydrogels. Three natural-origin polymers were selected for testing based on their gel-forming capabilities and biodegradability and biocompatibility: gelatin, carrageenan and sodium alginate [45]. Additionally, polymers with distinctly different characteristics were intentionally chosen. The physicochemical properties, solubility, consistency, and texture of gels depend on their origin and chemical structure. These differences play a crucial role in determining their potential applications. Gelatin is a water-soluble protein obtained by partial acidic or alkaline hydrolysis of animal collagen that forms thermoreversible gels. Gelatin has a similar chemical composition to native collagen, which consists of three α-chains arranged in a triple helix structure. This configuration offers an optimal geometry for inter-chain hydrogen bonding. Industrial gelatin is a mixture of various components, including α-chains, β-chains, and γ-chains. The molecular structure of gelatin predominantly features repeating sequences of GLY-X-Y triplets, where X is primarily proline and Y is predominantly hydroxyproline [46]. When heated, the triple helices are largely unraveled, and gelatin dissolves as random coils. When the solution is cooled, junction zones are formed by small segments from two or three polypeptide chains reverting to the collagen triple helix-like structure [47].The physical gel is influenced by extreme biodiversity due to the chemical composition of the native collagen, molecular weight distribution, solution properties resulting from the particular source of the gelatin [48]. It has good biodegradability, cell compatibility, and non-immunogenicity [49]. Carrageenan is an assortment of sulfated polyelectrolyte heteropolysaccharides extracted from certain species of red seaweed (*Rhodophyta*). Chemically, carrageenan is a linear sulfated galactan polymer consisting of alternating disaccharide repeating units of 3-linked β-D-galactopyranose (G units) and 4-linked α-D-galactopyranose (D units) or 4-linked 3,6-anhydro-α-D-galactopyranose (DA units). The three main types of carrageenan—κ, ι, and λ—differ primarily in their 3,6-anhydrogalactose content and the number of sulfate groups attached along the polymer chain [50]. It forms a thermoreversible gel as a function of temperature and ionic concentration [51]. So far, various mechanisms of gelation of this polysaccharide have been proposed. The most accepted model assumes that the gelation process involves the coil to helix transition, followed by the aggregation of helices and formation of a three-dimensional network. The extensive aggregation of κ-carrageenan helices yields to the formation of strong and hard gels with hysteresis in melting by heating. The gelation of carrageenan is a complex process that depends on the type and concentration of carrageenan and cations, and reaction temperature) [52]. Sodium alginate, a natural anionic polysaccharide derived from brown algae, is composed of 1–4 linked *α*-L-guluronic (G) and β-D-mannuronic (M) acid residues, with free hydroxyl (–OH) and carboxylate (–COO−) groups distributed along its backbone. This polymer is both pH- and electric-field-responsive. Below the pKa values of guluronic and mannuronic acid units (3.38 and 3.65, respectively), the carboxyl groups remain in their protonated form (–COOH). Above these pKa values, the carboxyl groups are ionized (–COO^−^) [53]. Sodium alginate forms a gel by binding divalent metal ions (Mg^2+^, Mn^2+^, Ca^2+^, Sr^2+^, Ba^2+^, Cu^2+^, and Pb^2+^); this ability is strongly dependent on the binding affinity of the polysaccharide to the metal and the chemical structure of an alginate [54]. Gelation and cross-linking of the alginate is achieved primarily by an exchange of sodium ions from the guluronic acids with the divalent cations, and the stacking of these guluronic groups by hydrogen bonds forms the characteristic egg-box structure [55]. Chemical and physical modifications of polymers are the subject of many scientific studies to improve their functionality [56].

The study aimed to assess the feasibility of using indirect treated plasma water (IPTW) for the preparation of hydrogels. We hypothesize that incorporating IPTW induces physicochemical changes in hydrogels, enhancing their practical applications.

## 2. Results and Discussion

### 2.1. Physicochemical Properties of Water Medium (DW and IPTW)

Physiochemical properties of distilled water (DW) and indirect-plasma-treated water (IPTW), including pH, oxidation-reduction potential (ORP) and conductivity (EC), are shown in Table 1. A lower pH, ORP and higher EC was noticed for IPTW. The results of pH of waters are in agreement with our previous findings [44]. The in-depth analysis of the water that we previously conducted revealed that the effect of plasma on the structure of water involves the resonance excitation of water aggregates. This resonance excitation leads to the breakdown of high molar mass aggregates into lower-molecular-mass ones. ESI MS spectra analysis showed that, after plasma exposure, there was a significant increase in the concentration of low-molecular solvated ions [M(H_2_O)]+ and [M(H_2_O)_2_]+ in all tested aqueous solutions. In contrast, the concentration of higher molecular mass ions, such as [M(H_2_O)_6-10_]+, solvated by water aggregates, decreased when compared to their concentrations in untreated water solutions. The obtained water exhibited lower pH and an unusual ability to absorb gases compared to the control sample. No increase in free radicals was observed. ORP is an important indicator for evaluating the redox potential of IPTW. High ORP values indicate an oxidative environment, whereas negative values are characteristic of a reductive environment. The ORP of IPTW was recorded at 314.90 mV, while 329.35 mV for DW sample. The obtained result confirms the absence of free radicals, typically present in PTW, such as NO, NO_2_−, and NO_3_− and other ions contributing to the enhancement of ORP as the powerful oxidizing agent [27]. The EC of untreated sample was found to be 1.43 mS/cm, while for IPTW it was equal to 2.43 mS/cm. Electrical conductivity reflects the capacity of water to conduct electrical current, which depends on the concentration of ions dissolved in it. The slight change in conductivity before and after plasma treatment are in agreement with Benija et al. [57] and Yelkin et al. [44].

### 2.2. Hydrogels pH Measurement

**Figure 1:**
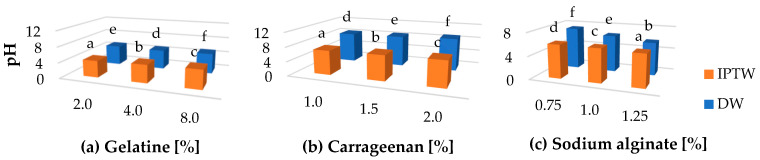
The effect of using indirect plasma treated water (IPTW) on the pH of (**a**) gelatine, (**b**) carrageenan, (**c**) sodium alginate hydrogels. a–f Different letters indicate significantly different groups determined by Duncan’s test (*p* < 0.05).

In Figure 1, the pH measurement results for polymer hydrogels prepared using distilled water and indirect treated plasma water (IPTW) are presented. The differences in the pH of alginate, carrageenan, and gelatin gels primarily stem from their chemical composition, gelation mechanisms, and the dissociation of functional groups present in the structures of these polymers. Alginate contains carboxyl groups, while carrageenan features sulfate groups, which contribute to its pH profile. Gelatin is inherently polyampholytic, meaning it contains both positive and negative charges distributed along its amino acid backbone. The overall properties of gelatin are determined by this charge distribution, as well as the presence of both polar and nonpolar amino acid side chains. The ionizable nature of these side chains makes gelatin sensitive to its environment, as its behavior can be significantly influenced by factors such as pH, ionic strength, and the presence of other substances in the surrounding medium [58,59,60]. For gelatin, carrageenan, and sodium alginate, the lowest pH values, specifically 4.26, 7.12, and 5.62, respectively, were observed in gels based on IPTW, while the highest values of pH were noticed for variants DW-G8 (5.16), DW-C2 (9.15), DW-A0.75 (7.35). The obtained results are consistent with our previous findings [2], where pH measurements were conducted on gelatin, carrageenan, and alginate hydrosols prepared using distilled and indirect plasma treated water (IPTW), also known as micro-clustered water. Previously, we assumed that the IPTW used in the process has similar properties to plasma-activated water (PAW). The decrease in pH of hydrosols with IPTW as potentially being caused by nitric and nitrous acids formed from the reactive species generated during plasma discharge was explained [61]. Based on the conducted research, we know that free radicals are not present in IPTW water [44]. The lower pH values of the hydrogels formed based on IPTW are due to its lower pH, as indicated by the results presented in Table 1. According to Yelkin et al. [44] an increase in the intensity of vibrations within water clusters may lead to the breaking of hydrogen bonds that connect ions in these clusters, resulting in a metastable state of charge separation. This process alters the structure of liquid water. Resonance amplification of the vibrations of structural elements in water can result in permanent changes to its physicochemical properties, including pH. The authors studied the effect of the interaction of electromagnetic fields resonance with water molecules, using the same plasma treatment method as we did and showed that as a result of such interaction, the pH of water decreases.

### 2.3. Fourier Transform Infrared Spectroscopy (FTIR)

**Figure 2:**
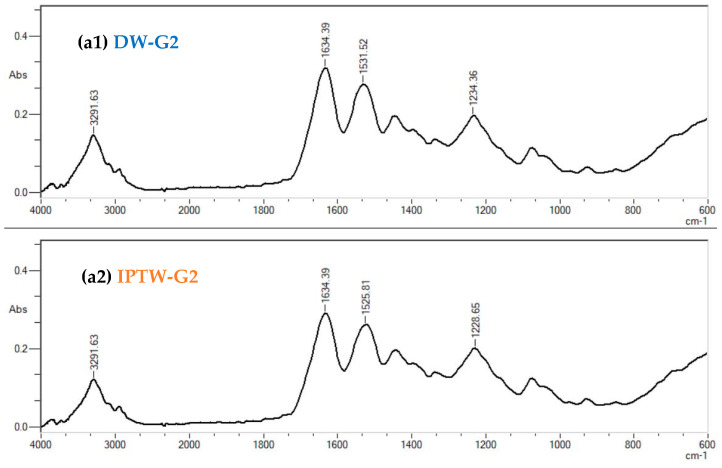

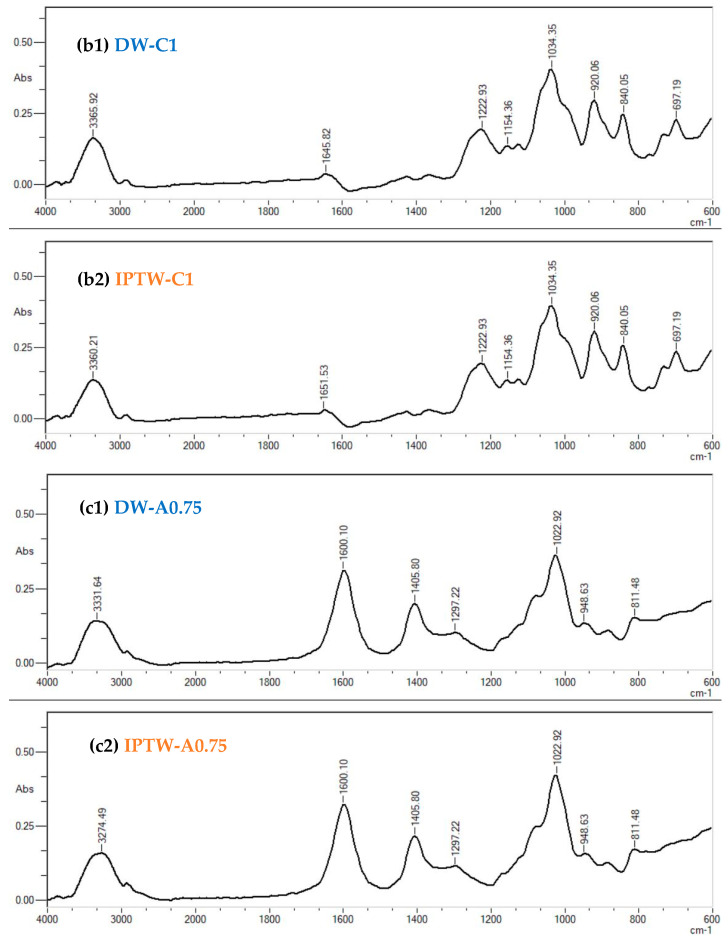
The FTIR spectra of: (**a**) 2% gelatin, (**b**) 1% carrageenan, (**c**) 0.75% sodium alginate in two variants of hydrogels: with distilled water (DW) and indirect-plasma-treated water (IPTW).

IR spectroscopy was performed to detect potential changes in the structure of gelatin, carrageenan, and alginate hydrogels resulting from the use of IPTW. The results are presented in Figure 2. When examining the spectroscopic characteristics of gelatin, Fourier Transform Infrared (FT-IR) spectroscopy primarily focuses on peptide-related amide bands, specifically amide A and amide I–III. In this study, broad peak centered around 3291 cm^−1^ attributed to the presence of hydrogen bond water and amide-A could be seen on Figure 2. The amide I band, linked to C=O stretching vibrations, is located at 1634 cm^−1^ in the control and tested sample. The amide II band, mainly derived from N–H bending and a minor contribution from C–N stretching vibrations, is detected at 1531 cm^−1^ in DW. In case of sample IPTW-G2, this band exhibits a 6 cm^−1^ shift to lower wavenumbers. Meanwhile, the amide III band, observed as weak peak at 1234 cm^−1^ (DW-G2) or 1228 cm^−1^ (IPTW-G2), dominated by C–N stretching, is complemented by N–H bending as well as contributions from backbone and side-chain vibrations [62].

In analysis of carrageenan FTIR spectra, the focus is on the fingerprint region (1225–700 cm^−1^). Several bands observed in this range are characteristic of polysaccharides, while others are linked to sulfate group vibrations. The band at 1222 cm^−1^ is associated with the asymmetric O=S=O vibration. The most intense band, located at 1034 cm^−1^, corresponds to the combination of C–O and S=O stretching modes. Another important feature is the absorption at 840 cm^−1^, attributed to the C–O–S linkage vibrations, which serves as a marker for the carrageenan conformation. This absorption at 840 cm^−1^ suggests the presence of the kappa-conformer. Additionally, two medium-intensity bands around 920 and 697 cm^−1^ are primarily attributed to the vibrations of C–O–C bridges, which are typical for polysaccharides [63].

In the case of sodium alginate, two primary spectral regions are typically examined. The first region, between 4000 and 2700 cm^−1^, is predominantly composed of O–H and C–H stretching vibrations. In the second region, two prominent bands at 1600 cm^−1^ and 1405 cm^−1^ are mainly attributed to the asymmetric and symmetric stretching vibrations of the carboxylate group. The second band may also show some contribution from C–OH deformation vibrations. Another significant band at 1022 cm^−1^ is primarily linked to C–O stretching vibrations. In the weak band region, particularly in the 950–750 cm^−1^ “fingerprint” region, typical for carbohydrates, the band at 948 cm^−1^ is assigned to the C–O stretching vibration of uronic acid residues, while the band at 811 cm^−1^ is characteristic of sodium alginate, attributed to mannuronic acid residues. A weak band at 1297 cm^−1^ is generally associated with C–C–H and O–C–H bending vibrations.

Generally, for gelatin, carrageenan, and sodium alginate hydrogels with DW and IPTW, the main spectral features are identified at similar wavenumbers. However, the change in peak height of the O-H stretching region (3250 cm^−1^) for gelatin, as well as the slight shift of this peak for carrageenan and alginate, may indicate the changes in the number of intramolecular hydrogen bonds of the hydroxyl groups, and thus indicate a different ability and way to bind water molecules in the hydrogels made with the use of DW and IPTW medium. Similar results were reported by Gebremical et al. [36] for starches mixed with PAW and by Hernandez-Perez et al. [64] and Sun et al. [65] for a different kind of starch treated by cold plasma. FTIR results indicated changes in functional groups related to the ability to bind water for starches mixed with PAW and treated with plasma. Ping et al. [66] and Zundel et al. [67] confirmed that water absorbed in hydrophilic polymers develops two types of hydrogen bonds: one corresponds to the water molecules directly attached to the to the active site of the polymer to form the first hydration layer (type-I attachment at 3250 cm^−1^), and the second to the molecules in the second hydration layer (type-II attachment at 3430 cm^−1^). Ping et al. [66] found that the OH stretching vibration band at ca. 3250 cm^−1^ in the poly(ethylene)-g-acrylic acid membranes (PE-g-AA) containing different concentrations of water, increases in intensity in direct proportion to the water content in PE-g-AA. The change in water content in the sample also influenced the mutual relationship in the peak heights observed at 3250 cm^−1^ and 3430 cm^−1^ and proved dynamic exchange of water molecules between sites and hydration layers. The lower peak intensity in the O-H stretching region and its shift to lower wavenumbers may reflects the change in power and type of interactions between absorbed water molecules and the IPTW polymer sites, which may be related to the presence of water aggregates and fewer direct connections of hydrogen bonds with the functional groups of polymers.

### 2.4. Rheological Measurements

**Figure 3:**
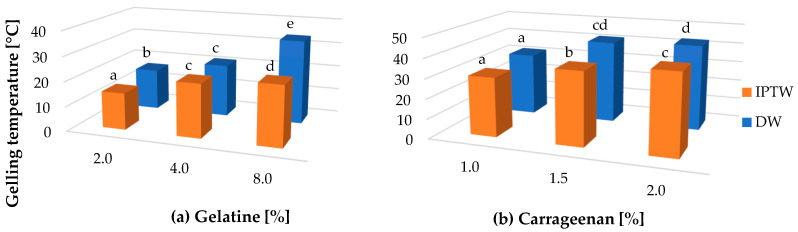
The effect of IPTW on the gelling temperature of (**a**) gelatin and (**b**) carrageenan. a–e: Different letters indicate significantly different groups determined by Duncan’s test (*p* < 0.05).

The gelling mechanism of gelatin is explained by the physical interactions amongst the molecules: electrostatic activity of the polypeptide chain and the formation of the hydrogen bond between the amino acid units, which participate in gel formation. Additionally, the helix is stabilized by the hydroxyl groups of hydroxyproline by inter-chain hydrogen bonding via bridging water molecules, as well as direct hydrogen bonding to the carbonyl group [68], whereas the gelling process of carrageenan solutions involves the coil-to-helix transition, followed by aggregation of double helices to form a spanning network [58]. Carrageenan and gelatin undergo a similar thermoreversible gelling mechanism, sol-to-gel phase transformation occurs as a result of cooling. In reference to the above the gelling temperature was recorded during cooling of hydrosol in temperature range from 60 °C to 10 °C. Because sodium alginate forms gels by crosslinking with divalent cations, it was not analyzed by this method. The gelling temperatures of both polymers are presented in Figure 3. It can be clearly seen that the gelation temperature increases with the polymer concentration depending on the type of polymer. The average gelling temperature of carrageenan in three different concentrations was 38.87 °C, while that of gelatin was 24.00 °C. On the other hand, gels prepared using IPTW are formed in significantly lower temperature than their references synthesized with deionized water. For gelatin and carrageenan IPTW hydrogels, 15.92% and 8.23% lower gelling temperatures were observed, respectively, in comparison to their reference samples. The results obtained from the FTIR analysis, indicating a change in water-binding capacity, confirm the alteration in the gelation temperature of gels containing IPTW. The observed direction of these changes can be mainly explained by the lowered pH of hydrosols prepared using IPTW [30]. Linus et al. [69] confirmed that lowering the pH of gelatin hydrosol from 4.4 to 4.2 results in a several-degree reduction in the gelation temperature. Depending on the type of gelatin, its isoelectric point (IP) is in the pH range of 4.7–5.2. At a pH value closer to IP, the net charges of polymer chains are minimal, and the effect of chain repulsion is limited, which favors the formation of hydrogen bonds and stabilization of the gel structure, which allows for gelation at a higher temperature. At a lower pH, the gelatin molecules are positively charged because the amino groups are protonated, which increases the repulsive forces between them and lowers the temperature needed to form a stable gel.

### 2.5. Texture Profiling Analysis

**Figure 4:**
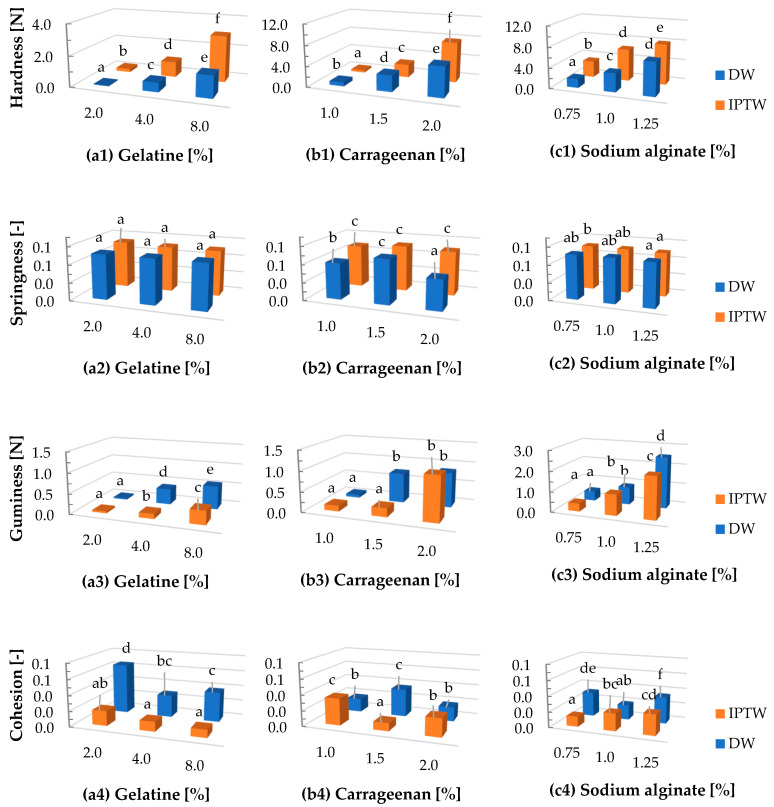

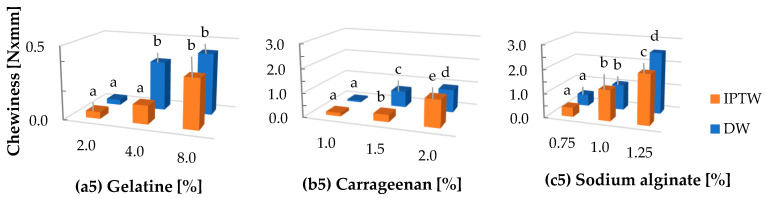
The influence of indirect plasma treated water (IPTW) on textural properties of (**a**) gelatine, (**b**) carrageenan, (**c**) sodium alginate hydrogels. a–f: Different letters indicate significantly different groups determined by Duncan’s test (*p* < 0.05).

The textural properties of the hydrogel are related to the network structure and strength of the polymer macromolecules involved in its formation. Generally, the difference in the texture profile of gelatin, carrageenan and sodium alginate hydrogels result from differences in their chemical composition, gelation mechanisms, cross-linking properties, interaction with water and pH [70,71]. The results of the texture profile analysis of hydrogels prepared based on IPTW and DW are shown in Figure 4. The use of IPTW significantly affected the hardness of the tested hydrogels. For both gelatin and sodium alginate, gels produced with IPTW exhibited greater hardness regardless of the polymer concentration. The same trend was also observed for carrageenan gels, but only at the highest polymer concentration of 2%. The IPTW-C2 variant demonstrated a hardness of 77.9, while the DW-C2 variant showed a hardness of 57.8. Differences in springiness, sometimes also defined as elasticity, were not noticeable in the gelatin samples. However, IPTW carrageenan gels and IPTW-A0.75 (1.0) displayed slightly higher values for this parameter compared to the control variants DW. The highest cohesiveness value for gelatin was measured for the DW sample at a concentration of 2% (0.7), while the highest cohesiveness for sodium alginate was recorded for the DW variant at the highest polymer concentration of 1.25, equal to 0.3. In the carrageenan samples, the highest cohesion value was measured for the IPTW-C1 variant (0.3) and the DW-C1.5 variant (0.4), where the differences were not statistically significant. The highest gumminess values for gelatin and sodium alginate were recorded in the DW variants at the highest polymer concentrations, whereas no statistically significant differences were found between DW and IPTW in the 2.0% carrageenan samples. The G4, C1.5, and A1.25 variants based on IPTW exhibited (Figure 4(a5,b5,c5)) lower chewiness compared to the control samples. The results can be explained by the fact that the plasma treatment may promote intermolecular or intramolecular cross-linking within the polymer molecules. This cross-linking increases the connectivity among polymer molecules, enabling the gel network to revert more readily to its original state after external stress, thereby improving the structural stability and elasticity of the gel [72]. The results obtained from FTIR analysis indicated a change in the water-banding capacity of tested hydrogels, which has a significant impact on their texture properties. Zuo et al. [73] and Gebremical et al. [36] confirmed that the application of water treated with plasma contributes to the increase in the hardness of gels.

### 2.6. Swelling Ratio

**Figure 5:**
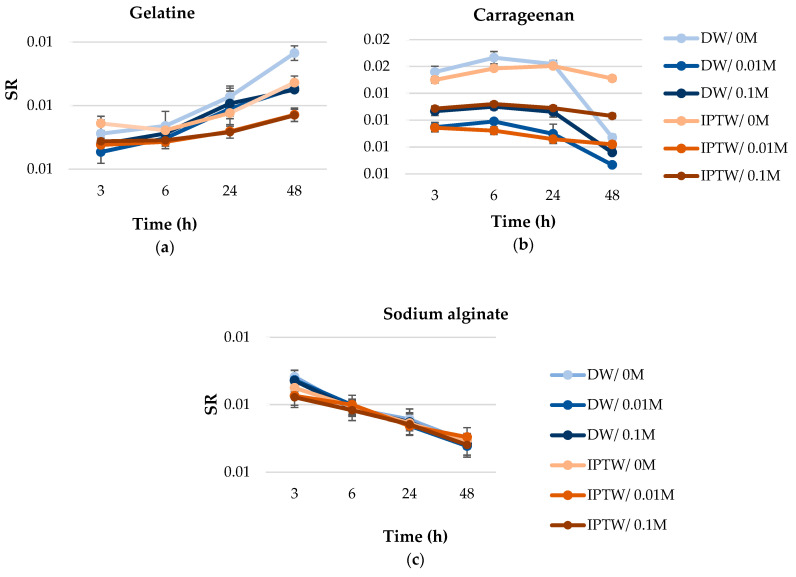
Swelling ratio (SR) of (**a**) 8% (*w*/*v*) gelatin, (**b**) 2.0% (*w*/*v*) carrageenan and (**c**) 1.25% (*w*/*v*) sodium alginate in two variants of the gels: DW, with deionized water; IPTW, indirect plasma treated water, submerged in different NaCl concentration (0 M, 0.01 M, 0.1 M).

Figure 5 presents the results of the swelling ratio (SR) measurements for gelatin, carrageenan, and alginate hydrogels in sodium chloride solutions of varying concentrations. Statistical analysis revealed a significant effect of the use of indirect-plasma-treated water (IPTW) in hydrogel production on the measured parameter. The differences in SR for gelatin, alginate, and carrageenan hydrogels based on IPTW compared to deionized water (DW) may be attributed to variations in polymer structure, water interactions, and the influence of salts [74,75]. This phenomenon can be explained by analyzing the properties of each biopolymer, their water-binding capacity, and the impact of IPTW on the polymer network. Gelatin hydrogels exhibit a gradual increase in SR over time, with hydrogels based on IPTW generally showing lower SR values compared to those based on DW. These differences are particularly noticeable in samples stored in water (0 M NaCl), where hydrogels based on deionized water show a greater increase in SR after 48 h. All gelatin hydrogel variants exhibited higher swelling ratios during storage in sodium chloride solutions (0.01 M, 0.1 M NaCl); however, this increase was more pronounced for hydrogels based on deionized water. After 3 h of analysis, the lowest SR was observed in the gelatin hydrogels based on deionized water, with values of 1.05, 1.08, and 1.11, respectively, while the highest SR was found in the IPTW variants (1.08, 1.09, 1.14). During the course of the study, this trend changed. After 48 h, the IPTW samples had a significantly lower SR (IPTW/0.01 1.17; IPTW/0.1 1.17; IPTW/0 1.27) compared to the control variants (DW/0.01 M NaCl 1.25; DW/0.1 M NaCl 1.25; DW/1.37). Gelatin, as a protein, forms a network structure that binds water mainly through hydrogen bonds. The swelling ratio of gelatin hydrogels depends on the network structure and the availability of free sites for water binding. Plasma treatment may affect the hydrogel structure by reducing the number of sites available for water absorption, resulting in lower SR values [76]. The impact of using IPTW for hydrogel production on the polymer’s water-binding capacity was confirmed by FTIR analysis. This may be related to the different structure of water clusters, which limits the mobility of water molecules and their ability to interact with the gelatin polymer. In the case of alginate hydrogels, SR decreases with the duration of the analysis. IPTW hydrogels have a higher SR in the initial stages (3–6 h), but the decrease in swelling capacity is more rapid compared to DW hydrogels. After 3 h, the highest SR values were observed in IPTW/0.01 M NaCl (0.92) and IPTW/0.1 M NaCl (0.92), while the lowest water absorption capacity was recorded for the DW/0 M NaCl variant. After 48 h, no statistically significant differences were found between DW and IPTW hydrogels. NaCl concentration affects water retention in alginate, consistent with previous reports on alginate hydrogel degradation in salt solutions [77]. Indirect plasma treated water may promote faster water loss, suggesting more dynamic ion–polymer interactions in the presence of IPTW molecules. A statistically significant, opposite relationship of the effect of IPTW on the swelling ratio of hydrogels was observed in carrageenan samples. The IPTW variants, after 48 h of analysis, showed higher SR values of 0.44 (0 M NaCl), 0.15 (0.1 M NaCl), and 0.27 (0.01 M NaCl), respectively. The differences in the different swelling ratios of carrageenan, gelatin and sodium alginate are in agreement with our previous findings [68]. Swelling and disintegration prove the hydrophilic nature of carrageenan [78]. Carrageenan, a polysaccharide, has an anionic structure that allows for strong water binding in the presence of ions such as Na⁺ and K⁺, which promotes swelling [79]. IPTW may enhance the ability of carrageenan to swell by facilitating better organization of water molecules around the anionic sulfate groups of carrageenan. The water structure is an important factor governing the physical and chemical properties of hydrogels [23].

### 2.7. Scanning Electron Microscopy

**Figure 6:**
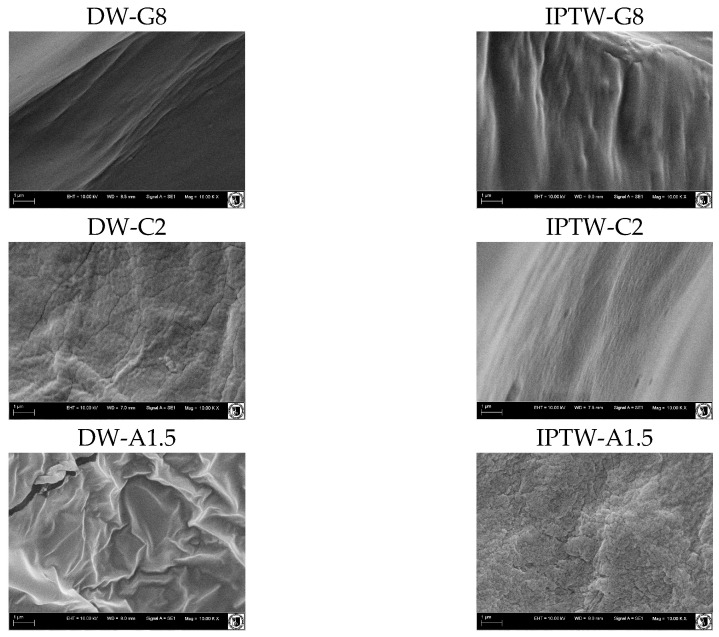
SEM images of the surface of hydrogels (1000× magnitude).

The morphology of gelatin, carrageenan and sodium alginate hydrogels prepared based on deionized and indirect plasma treated water (IPTW) is presented in Figure 6. There are noticeable differences in the microstructure of the tested hydrogels. The control samples of gelatin (DW-G8) and sodium alginate (DW-A1.5) hydrogels generally display a smoother, more uniform texture, while the samples prepared based on IPTW (IPTW-G8, IPTW-A1.5) have more uneven and fibrous structure. The opposite effect was observed for carrageenan gels, which confirms the differences in the characteristics of the polymers [80,81] and their reaction with IPTW. The results suggest that IPTW influences the structural arrangement of the polymer matrix, making it more heterogeneous in the case of gelatin and sodium alginate and more homogenous in the case of carrageenan. Differences in porosity might also indicate that the use of plasma influences on cross-linking density. According to Ma et al. [82] the increased hydrogen content in plasma-activated water influenced the molecular hydrogen bonding within the starch paste, while the acidic properties of PAW partially eroded the wheat starch granules. This resulted in the formation of a more robust gel network and a denser structural arrangement. Zeng et al. [83] observed that a compact and heterogeneous structural network enhances the mechanical strength of starch gels, which is consistent with our texture test results.

### 2.8. Color Measurement

The results of the color measurements of gelatin, carrageenan, and alginate hydrogels are presented in Table 2. Based on statistical analysis, significant differences were observed in the color of samples prepared with deionized water (DW) and indirect-plasma-treated water (IPTW). In the case of gelatin hydrogels, the use of IPTW significantly increased L* values, indicating greater brightness compared to hydrogels prepared with deionized water. The L* parameter for IPTW samples ranged from 51.29 to 56.56, while for DW variants, it ranged from 43.22 to 44.41. The highest L* value was recorded for the IPTW-G2 sample, reaching 56.56, which may indicate the highest transparency. Additionally, a shift in the a* value (red–green spectrum) was noted, with positive values in DW variants and negative values in IPTW samples, indicating a greener hue. The highest a* values, 0.83 and 0.88, were recorded for DW-G2 and DW-G4 samples, respectively, while the lowest was −3.17 for IPTW-G8. Similarly, a significant difference in the b* parameter, representing the blue-yellow spectrum, was observed, indicating a reduction in yellowness in IPTW hydrogels. The lowest b* values were recorded for IPTW-G8 and IPTW-G2 at −1.34 and −1.11, respectively, compared to 4.31 for DW-G8. The color differences described above are reflected in the ΔE*ab value, which was significantly higher for IPTW variants. The largest color differences were observed for IPTW-G4 (9.30) and IPTW-G8 (9.57). The use of IPTW in carrageenan gel production also significantly affected the color of the materials studied. A clear increase in the L* parameter was observed, indicating greater brightness in IPTW variants. The L* values ranged from 44.09 to 45.45 for DW samples and from 50.62 to 51.48 for IPTW. Negative a* values were recorded for IPTW variants, which, similar to gelatin hydrogels, indicate a greater contribution of the green hue. The lowest a* value was measured for the IPTW-C1 sample (−2.25), while the highest was for the DW-C1, DW-C1.5, and DW-C2 samples, ranging from 1.17 to 1.20. The b* values in carrageenan hydrogels indicated a reduction in yellowness in IPTW samples, especially in IPTW-C2 and IPTW-C1.5, where the b* values were −1.10 and −1.36, respectively, compared to 2.13 in DW-C2. The ΔE*ab results clearly indicate a significant difference in color between the control (DW) and experimental (IPTW) variants. A similar trend in color change was observed in the case of alginate hydrogel samples. The L* values of the IPTW variants were statistically higher (51.66, 52.63, 53.80) than in DW (44.49, 45.70, 46.64). The indirect-plasma-treated-water-based samples also had a higher contribution of green color, with the a* parameter ranging from −2.05 to −1.97, while in deionized water variants, it ranged from 1.43 to 1.56. The use of IPTW contributed to a shift in the b* parameter toward a greater contribution of blue tones, with values ranging from −5.64 to −4.97 for IPTW, and from −1.37 to −0.73 for DW. The ΔE*ab results clearly indicate a color difference between hydrogels based on IPTW and those based on deionized water. The highest ΔE*ab values were measured for the IPTW-A0.75 and IPTW-A1.25 variants, at 9.69 and 9.39, respectively. The results are consistent with those obtained by Zuo et.al [73]. The authors investigated the effect of plasma-activated water (PAW) on corn starch. As a result of applying PAW the color of the tested material changed, particularly with a noticeable increase in the brightness of the samples, as indicated by an increase in the L* parameter. The increase in the ΔE*ab parameter as a result of using IPTW may stem from changes in the polymer structure due to plasma modification. Changes in surface roughness may affect light reflection [84]. IPTW has a lower pH compared to distilled water, as indicated by the result presented in Table 1. The use of IPTW contributed to the reduction in the pH of the hydrogels. The change in the pH of the hydrogels may modify the net charge of molecules. This alternation can, in turn, affect the attractive and repulsive forces between molecules, as well as the interactions between the molecules and the solvent, commonly referred to as the hydration properties. As a result, the physical and chemical properties of the hydrogels, including their color, may be altered [71].

## 3. Materials and Methods

### 3.1. Material

In this study, Alginate FD 125, sourced from *Laminaria digitata* (molecular weight 140 kDa, particle size max. 2% > 620 µm, M:G ratio = 1.2), was procured from Dupont GRINSTED^®^ in Grinsted, Denmark. Gelatin (CAS-No.: 9000-70-8) derived from porcine skin (180 Bloom) was acquired from Weishardt in Graulhet, France, ҝ-carrageenan (CAS-No.: 11114-20-8) sourced from *Euchema cottoni* was obtained from Regis in Kraków, Poland, calcium chloride was supplied by P.P.H. ‘STANLAB’ Sp. J., Lublin, Poland.

### 3.2. Plasma Treatment

Water treatment was conducted utilizing the apparatus outlined in [44]. The treatment duration lasted for 15 min under a pressure of 10 Pa and a discharge power of 8.5 W. The pressure level in the plasma reactor was maintained dynamically by regulating the amount of fresh air entering the reactor vessel while it is pumped out by a standard 2-stage vacuum pump. As mentioned earlier the portion of the so introduced water vapor is essential from the point of view of ‘coherization’ of the plasma-generated wide spectrum of frequencies forming the noise.

### 3.3. Preparation of Hydrogels

Hydrogels were meticulously crafted through the dissolution of gelatin (G), carrageenan (C), and sodium alginate (A) in either distilled (DW) or indirect plasma treated water (IPTW). To achieve this, gelatin and carrageenan solutions underwent a heating process up to 60 °C, accompanied by stirring at a rate of 300 rpm using an IKA RW 20 digital stirrer for 10 min. After liquefaction, the solutions were kept at room temperature to form a gel and then stored at 4 °C for 24 h. Meanwhile, sodium alginate hydrogels were prepared by dissolving sodium alginate in DW or IPTW at room temperature with an extended stirring period of 30 min to ensure optimal integration. Obtained hydrosols were poured into semi-permeable cellulose casings and immersed in 0.5 M CaCl_2_ solution, acting as a cross-linking agent, to form a gel and subsequently stored at 4 °C for 24 h [85]. The composition of the tested samples is presented in Table 3.

### 3.4. Physiochemical Properties of Water Medium (DW and IPTW)

The pH, oxidation-reduction potential (ORP) and electrical conductivity (EC) of DW and IPTW were measured using a pH/mV/ISE Meter (Seven Multi™ model S40, Mettler Toledo, Warsaw, Poland) equipped with a pH electrode (Inlab Routine Pro, Mettler Toledo, Greifensee, Switzerland), ORP electrode (Inlab Redox Pro, Mettler Toledo, Greifensee, Switzerland) and conductivity electrode (InlabLab 731, Mettler Toledo, Greifensee, Switzerland), respectively.

### 3.5. Hydrogels Characterization

#### 3.5.1. pH Measurement

pH measurements within the geometric centers of the gels were conducted employing an electrode linked to a pH meter, specifically the HI 99161 model (Hanna instruments, Ann Arbor, MI, USA) [85].

#### 3.5.2. Fourier Transform Infrared Spectrometry (FTIR)

Infrared spectra were registered in a Shimadzu spectrometer (ATI Mattson, Kyoto, Japan). The transmission spectra were collected at 2 cm^−1^ resolution and by 64 scans, directly on polymer material with a golden bridge reflexion apparatus. Before analysis, the gels were frozen at −80 °C (24 h) and then lyophilized (vacuum 0.060 mBar/−48 °C) for 2 days and stored in a desiccator until analysis.

#### 3.5.3. Rheological Measurements

Gelation temperature was determined through the utilization of a Haake RheoStress 6000 rheometer (Thermo Scientific, Karlsruhe, Germany), operated in oscillatory mode with a strain of 5% and a frequency of 1 Hz. These conditions were carefully verified to ensure operation within the linear viscoelastic region. The storage modulus (G’) and loss modulus (G”) were recorded as a function of temperature. For the assessment, one milliliter of gelatin or carrageenan hydrosols was applied onto the measurement plate using a cone/plate geometry with a 0.52 mm cone. To prevent hydrosol evaporation, paraffin oil was applied around the measured area. The results of G’ and G” were obtained during two stages: the cooling phase from 75 °C to 15 °C and the heating phase from 15 °C to 75 °C. Temperature ramps of 1.2 °C/min were applied. The equilibrium determination of G’ = G” for evaluating the variability of both modules with temperature elucidated the conditions of sol-gel phase transition (stage I) and gel-sol transition (stage II). Gelation (Tg) temperature of hydrosols were determined based on the intersection point of the curves. All tests were executed using a cone sensor (C60/1 Ti L, Thermo Scientific, Karlsruhe, Germany) and a measuring plate (TMP60 Steel 18/8 Thermo Scientific, Karlsruhe, Germany) in CS mode. The measuring device was operated with RheoWin Job Manager version 4.00 software (Haake, Vreden, Germany).

#### 3.5.4. Texture Profile Analysis

The Texture Profile Analysis (TPA) procedure was conducted at room temperature using the Zwick Roell Z010, type: Z6FD1 (Zwick Roell, Ulm, Germany), featuring a head capable of measuring loads up to 100 N. Gel samples were meticulously positioned between parallel flat plate fixtures, and affixed to a TA.XT2 Texture Analyzer (Stable Micro Systems, Surrey, UK) interfaced with a microcomputer. All measurements were executed on gels that had been allowed to equilibrate to room temperature. The gels underwent a dual compression at 70% deformation, with a subsequent relaxation time of 30 s. The ensuing parameters were quantified as outlined in [86]:

- Hardness [N]: representing the maximum force required to compress the sample.
- Springiness [-]: defined as the distance the sample rebounded during the second compression to the peak force, divided by the initial sample height.
- Cohesion [-]: characterized by the ratio of the area under the first and second compressions.
- Gumminess [N]: computed as the product of hardness and cohesiveness.
- chewiness [N × mm]: calculated by multiplying gumminess with springiness.

#### 3.5.5. Swelling Ratio

The hydrogels were carefully weighed and immersed in solutions of 0 (distilled water), 0.01, and 0.1 M NaCl. At predetermined intervals, the swollen gels were extracted from the swelling medium, gently dried with filter paper, and re-weighed before being re-immersed in the respective bath. The swelling ratio of the hydrogels was computed using the following equation:

(1) $$SR=\frac{W_{s}}{W_{o}}$$

where W_s_ is the weight of a swollen hydrogel and W_o_ is the weight of a dried hydrogel.

#### 3.5.6. Scanning Electron Microscopy

The microstructures of gelatin, carrageenan, and sodium alginate hydrogels were analyzed using Scanning Electron Microscopy (SEM) using the EVO LS15 ZEISS Scanning Electron Microscope (Zeiss, Jena, Germany). Test samples were carefully sectioned into smaller pieces of approximately 0.5 mm² and coated with a 10 nm-thick layer of gold for 150 s using a sputter coater Scancoat 6 (Edwards, London, UK). Subsequently, each coated sample underwent examination under a voltage of 20 kV.

#### 3.5.7. Color Measurement

The hydrogels’ colors were assessed utilizing a reflective colorimeter, MINOLTA CR-400, along with a CR-A33d Light Projection Tube (ø 22 mm disc, Konica Minolta, Osaka, Japan), configured at C illuminant and 2 standard observer settings. Before each series of measurements, the chroma meter underwent calibration using a white ceramic plate (White Calibration Plate CR-A33a, Konica Minolta, Osaka, Japan) with the following coefficient values: Y = 93.8, x = 0.3158, y = 0.3323. The CIELAB color scale was employed for color measurement, spanning from L* = 0 (black) to L* = 100 (white), −a* (greenness) to +a* (redness), and −b* (blueness) to +b* (yellowness). Color differences (ΔE*ab) between the control (D gels) and experimental samples (MC) were determined using the following equation:

(2) $${∆E}^{*}ab=\sqrt{{\left(L^{*}sample-L^{*}control\right)}^{2}+{\left(a^{*}sample-a^{*}control\right)}^{2}+{\left(b^{*}sample-b^{*}control\right)}^{2}}$$

### 3.6. Statistical Analysis

Every experiment was conducted in triplicate to ensure robustness and reliability. Statistical analysis was carried out employing univariate and multivariate Analysis of Variance (ANOVA) techniques utilizing Statistica 12 (StatSoft, Kraków, Poland). To discern significant differences among mean values, the Duncan Test was applied with a confidence level set at *p* < 0.05.

## 4. Conclusions

The use of plasma to treat water is a technology that is finding increasingly wider application in various industries due to the unique physicochemical properties that are obtained as a result of such interaction. The plasma treatment methods described in the literature are mainly based on water structuring with the simultaneous generation of reactive oxygen and nitrogen species (RONS) [87]. However, it is worth emphasizing that the generation of free radicals may be perceived as an undesirable effect and may limit the use of plasma-treated water. The new method of obtaining water by indirect interaction with plasma (IPTW) described in this article allows for changing the physical properties of water without generating free radicals [44]. The use of IPTW to produce hydrogels contributed to changes in their physicochemical properties. Hydrogels produced using IPTW water exhibited a different water-binding capacity and lower pH, which significantly influenced their properties. IPTW hydrogels were characterized by higher hardness, lower gelation temperature, lighter color. The use of IPTW also significantly affected the microstructure of the tested materials. Changes in the physicochemical properties of hydrogels produced using IPTW may be of significant importance in expanding their application possibilities.

## Figures and Tables

**Table 1:** Physicochemical properties of distilled water before and after indirect plasma treatment.

Type of Water	pH	ORP [mV]	EC [mS/cm]
DW	7.20 ^b^ ± 0.11	329.35 ^b^ ± 5.17	1.43 ^a^ ± 0.13
IPTW	6.78 ^a^ ± 0.11	314.90 ^a^ ± 5.61	2.43 ^b^ ± 0.29

^a,b^: Different letters indicate significantly different groups determined by Duncan’s test (*p* < 0.05).

**Table 2:** The effects of the indirect-plasma-treated water (IPTW) on the color of hydrogels.

Variant	L*	a*	b*	∆E*ab
DW-G2	43.33 ± 0.64 ^a^	0.83 ± 0.10 ^d^	1.35 ± 0.35 ^c^	0.00 ± 0.00 ^a^
DW-G4	43.22 ± 0.58 ^a^	0.88 ± 0.13 ^d^	1.37 ± 0.41 ^c^	0.00 ± 0.00 ^a^
DW-G8	44.41 ± 0.67 ^b^	0.58 ± 0.05 ^c^	4.31 ± 0.36 ^d^	0.00 ± 0.00 ^a^
IPTW-G2	56.56 ± 0.69 ^d^	−2.66 ± 0.29 ^a^	−1.11 ± 0.56 ^b^	5.70 ± 0.32 ^b^
IPTW-G4	51.29 ± 0.62 ^c^	−2.64 ± 0.11 ^a^	−1.61± 0.25 ^a^	9.30 ± 0.21 ^c^
IPTW-G8	52.69 ± 0.73 ^c^	−3.17 ± 0.21 ^b^	−1.34 ± 0.42 ^b^	9.57 ± 0.20 ^c^
DW-C1	45.45 ± 0.83 ^b^	1.19 ± 0.09 ^a^	−0.45 ± 0.26 ^c^	0.00 ± 0.00 ^a^
DW-C1.5	44.09 ± 0.95 ^a^	1.17 ± 0.16 ^a^	0.73 ± 0.42 ^d^	0.00 ± 0.00 ^a^
DW-C2	44.28 ± 0.97 ^a^	1.20 ± 0.15 ^a^	2.13 ± 0.31 ^e^	0.00 ± 0.00 ^a^
IPTW-C1	51.48 ± 0.74 ^d^	−2.25 ± 0.14 ^c^	−3.22 ± 0.94 ^b^	7.47 ± 0.21 ^b^
IPTW-C1.5	50.67 ± 0.60 ^c^	−2.31 ± 0.14 ^bc^	−1.36 ± 0.76 ^a^	7.73 ± 0.13 ^b^
IPTW-C2	50.62 ± 0.68 ^c^	−2.39 ± 0.08 ^b^	−1.10 ± 0.72 ^a^	7.97 ± 0.22 ^b^
DW-A0.75	44.49 ± 0.77 ^a^	1.52 ± 0.15 ^b^	−1.15 ± 0.94 ^b^	0.00 ± 0.00 ^a^
DW-A1	45.70 ± 0.89 ^a^	1.43 ± 0.12 ^b^	−1.37 ± 0.82 ^b^	0.00 ± 0.00 ^a^
DW-A1.25	46.64 ± 0.91 ^a^	1.56 ± 0.11 ^b^	−0.73 ± 0.86 ^b^	0.00 ± 0.00 ^a^
IPTW-A0.75	52.63 ± 0.99 ^b^	−2.05 ± 0.21 ^a^	−5.02 ± 0.86 ^a^	9.69 ± 0.21 ^c^
IPTW-A1	51.66 ± 0.99 ^b^	−1.97 ± 0.07 ^a^	−4.97 ± 0.97 ^a^	7.75 ± 0.27 ^b^
IPTW-A1.25	53.80 ± 0.95 ^b^	−2.02 ± 0.28 ^a^	−5.64 ± 0.57 ^a^	9.39 ± 0.22 ^c^

L*, a*, b* – color coordinates in the CIELAB space (lightness, green-red hue, blue-yellow hue), ΔE*ab – color difference. ^a–e^: Different letters indicate significantly different groups determined by Duncan’s test (*p* < 0.05).

**Table 3:** The composition of hydrogels.

Hydrocolloid	[%]	Water	
		Distilled (DW)	Indirect Plasma Treated (IPTW)
		Run Code Letters	
**Gelatin (G)**	2.0	DW-G2	IPTW-G2
	4.0	DW-G4	IPTW-G4
	8.0	DW-G8	IPTW-G8
**Carrageenan (C)**	1.0	DW-C1	IPTW-C1
	1.5	DW-C1.5	IPTW-C1.5
	2.0	DW-C2	IPTW-C2
**Sodium alginate (A)**	0.75	DW-A0.75	IPTW-A0.75
	1.0	DW-A1	IPTW-A1
	1.25	DW-A1.25	IPTW-A1.25

## Data Availability

The data presented in this study are available on request from the corresponding author.
